# An integrated device for fast and sensitive immunosuppressant detection

**DOI:** 10.1007/s00216-021-03847-x

**Published:** 2021-12-22

**Authors:** Sara Tombelli, Cosimo Trono, Simone Berneschi, Chiara Berrettoni, Ambra Giannetti, Romeo Bernini, Gianluca Persichetti, Genni Testa, Guillermo Orellana, Francesca Salis, Susanne Weber, Peter B. Luppa, Giampiero Porro, Giovanna Quarto, Markus Schubert, Marcel Berner, Paulo P. Freitas, Susana Cardoso, Fernando Franco, Vânia Silverio, Maria Lopez-Martinez, Urs Hilbig, Kathrin Freudenberger, Günter Gauglitz, Holger Becker, Claudia Gärtner, Mark T. O’Connell, Francesco Baldini

**Affiliations:** 1 Institute of Applied Physics “Nello Carrara”, CNR-IFAC, Via Madonna del Piano 10, 50019 Sesto Fiorentino, Italy; 2 Institute for Electromagnetic Sensing of the Environment, CNR-IREA, Via Diocleziano 328, 80124 Napoli, Italy; 3 Department of Organic Chemistry, Faculty of Chemistry, Universidad Complutense de Madrid, 28040 Madrid, Spain; 4 Institute of Clinical Chemistry and Pathobiochemistry, Klinikum rechts der Isar, Technische Universität München, Marchioninistrasse 15, 8000 Munich, Germany; 5 Datamed Srl, Via Grandi 4/6, 20068 - Peschiera Borromeo, Milan, Italy; 6 Institute for Photovoltaics and Research Center SCoPE, University of Stuttgart, 70569 Stuttgart, Germany; 7 Innovative Pyrotechnik GmbH, Steinwerkstraße 2, 71139 Ehningen, Germany; 8 Instituto de Engenharia de Sistemas e Computadores-Microsistemas e Nanotecnologias, R.Alves Redol 9, 1000-027 Lisbon, Portugal; 9 Institute for Physical and Theoretical Chemistry, Eberhard Karls University, Auf der Morgenstelle 18, 72076 Tübingen, Germany; 10 microfluidic ChipShop GmbH, Stockholmer Str. 20, 07747 Jena, Germany; 11 Cornel Medical Limited, 17 Church Walk, St Neots, Cambridgeshire, PE19 1JH UK

**Keywords:** Immunosuppressant, POCT, Fluorescence, Cyclosporine A, Mycophenolic acid

## Abstract

The present paper describes a compact point of care (POC) optical device for therapeutic drug monitoring (TDM). The core of the device is a disposable plastic chip where an immunoassay for the determination of immunosuppressants takes place. The chip is designed in order to have ten parallel microchannels allowing the simultaneous detection of more than one analyte with replicate measurements. The device is equipped with a microfluidic system, which provides sample mixing with the necessary chemicals and pumping samples, reagents and buffers into the measurement chip, and with integrated thin film amorphous silicon photodiodes for the fluorescence detection. Submicrometric fluorescent magnetic particles are used as support in the immunoassay in order to improve the efficiency of the assay. In particular, the magnetic feature is used to concentrate the antibody onto the sensing layer leading to a much faster implementation of the assay, while the fluorescent feature is used to increase the optical signal leading to a larger optical dynamic change and consequently a better sensitivity and a lower limit of detection. The design and development of the whole integrated optical device are here illustrated. In addition, detection of mycophenolic acid and cyclosporine A in spiked solutions and in microdialysate samples from patient blood with the implemented device are reported.

**Graphical abstract:**

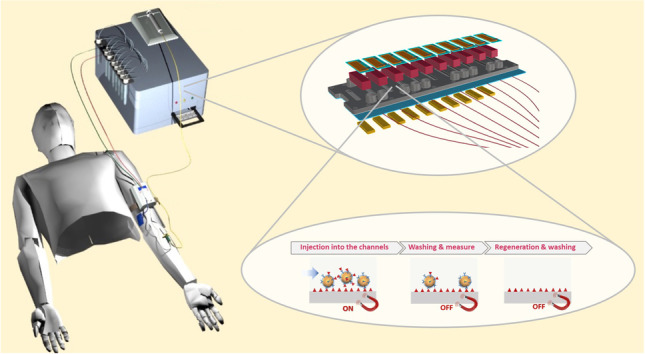

**Supplementary Information:**

The online version contains supplementary material available at 10.1007/s00216-021-03847-x.

## Introduction

Point of care testing (POCT) devices measure analytes of clinical interest close to the patient [1–3], providing physicians the possibility to achieve the correct diagnosis or to administer the right therapy in a relatively short time and their use is more and more diffusing with the approval of the competent authorities responsible for overseeing the enforcement of medical device regulations. They are generally able to perform single-shot measurements on different biological matrices (blood, urine, saliva, etc.) while a frequent/near-continuous measurement remains a challenging task in the clinical practice, so far confined to laboratory and at prototype level, with the exclusion of rare exceptions such as the detection of glucose in diabetic patients. The advent of personalised medicine has shown the importance, if not the compelling need, to tailor the right therapeutic strategy for the right person at the right time, and therapeutic drug monitoring (TDM) with POCT devices is becoming more and more important in many pathologies, very often associated to the need of frequent/near-continuous measurement of the analyte of interest [4–6].

Immunosuppressant monitoring after organ transplantation is a mandatory issue for the life of transplanted patients: if levels of immunosuppressive drugs are too high, the inhibition of the immune system is excessive and the patient may be unable to fight off infections, while if the dosage is too low there is a danger of organ rejection. Drugs of interest are chemicals with a narrow therapeutic window, which regularly pose considerable problems in initial and ongoing dosing [7, 8]. These drugs are often subject to polymorphic metabolism with considerable inter- and intra-individual variability requiring TDM for personalised dosage [9]. As these drugs are highly sequestered in erythrocytes and leukocytes, and also reversibly bound to plasma proteins, their concentration is routinely measured nowadays in haemolysed whole blood samples, by means of laboratory techniques, such as liquid chromatography–tandem mass spectrometry (LC-MS/MS) and fluorescence or chemiluminescence immunoassays [8, 10, 11]. This implies that TDM is usually performed with only one blood sample per dosing interval taken either prior or 1–2 h after drug administration. The drug concentration is subsequently evaluated in the laboratory and barely available just before the next dose. In the best case, this approach provides information on the trough and peak levels, which are the lowest and highest concentration reached by a drug before the next dose is administered, respectively. Recent studies have shown that more accurate clinical indication is given by the area under the curve (AUC) of the drug concentration vs time, the determination of which would need a frequent — ideally continuous — monitoring, as this value is better correlated with both the efficiency and side-effects of the administered drug therapy than the trough level [12–14].

On this basis, it is clear that a device capable to measure frequently immunosuppressants close to transplanted patients is a strong plea of physicians in transplantation surgery for the determination of the right dosage of the administered drugs in the first period after transplantation, increasing the number of relevant or evolving clinical parameters to be analysed with POCT devices [15]. Among the typical immunosuppressants, the calcineurin inhibitors cyclosporine A [16] and tacrolimus (FK 506) [17, 18] are of particular interest, as well as mycophenolic acid [19, 20], which inhibits an enzyme needed for the growth of T- and B-cells. The device must be also able to perform simultaneous measurement of more than one analyte, since the administration of a mixture of immunosuppressants instead of a single drug is often the followed protocol in the post-operative treatment of transplanted patients [21, 22]. It is also important to observe that the free fraction of immunosuppressants circulating in blood has been recognised the responsible for their pharmacological activity, as well as for associated side effects [23]. The reason why the free fraction is not addressed by TDM not only lies in the lack of relevant reference values available [24] but also because of its very low concentration, much lower (just a few %) than the total concentration in the blood, i.e. down to ng/mL or less. In a recent paper [25], intravascular microdialysis has been demonstrated a reliable technique to extract the free fraction of immunosuppressants paving the way towards quasi-continuous therapeutic drug monitoring in transplanted patient; this can provide a more effective control of the drug uptake by the transplanted patients in a standard routine, unthinkable to be performed nowadays, and quickly allowing the correct dosing of the administered drugs, which is essential for the therapy outcome and the patient’s safety.

The need for quasi-continuous/frequent measurements makes it necessary to adopt suitable strategies to reduce as much as possible the assay time. Magnetic particles have been shown to be an efficient tool to accelerate the assay time while maintaining high sensitivity, providing the possibility to reduce the incubation time and preserving high binding efficiencies [26, 27]. Furthermore, the addition of a fluorescent label makes their easy integration in optical bioassays possible [28], which is an important aspect to be considered since optical biosensors are playing a relevant role in TDM [29, 30].

Having in mind to fill the gap nowadays present in TDM-POCT devices, the present paper describes a compact optical device capable to perform a fluorescence-based immunoassay, using a disposable plastic chip where the assay takes place for the determination of immunosuppressants. The particular structure of the chip with ten parallel microchannels allows the simultaneous detection of more than one analyte with replicate measurements. The device is equipped with microfluidic circuitry, which handles the sample mixing with the necessary chemicals using a second suitable chip and pumping it into the measurement chip, and with integrated thin film amorphous silicon photodiodes for the fluorescence detection. Submicrometric fluorescent magnetic particles are used as support in the immunoassay in order to improve the efficiency of the assay. In particular, the magnetic feature is used to concentrate the antibody onto the sensing layer leading to a much faster implementation of the assay, while the fluorescent feature is used to increase the optical signal leading to a larger optical dynamic change and consequently a better sensitivity and a lower limit of detection. For the purpose to perform quasi-continuous/frequent measurements over a period of several hours on the same patient with only one chip, the regeneration of the chip was also implemented. In this way, the developed chip could be considered as a ‘single-patient’ chip, close to the concept of single-use chip, which is usually required in clinical chemistry. The use of the device with microdialysate samples allowing continuous sampling for 12–48 h would ease the assessment of the AUC, which is considered increasingly important for pharmacokinetic monitoring of immunosuppressive drugs [14].

## Experimental

### The optical device

**Fig. 1:**
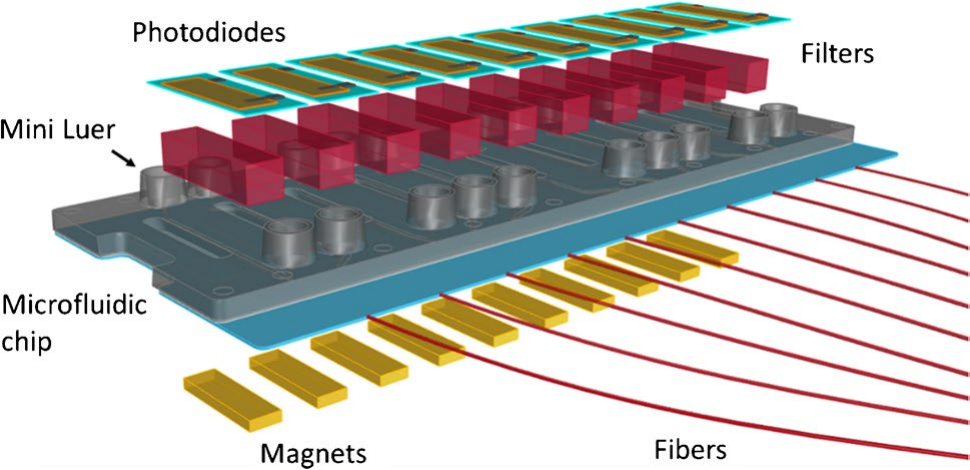
Exploded view of the core of the POCT system

The device was composed of an optoelectronic system which comprised an excitation and detection module and a fluidic circuit for handling the sample and it was equipped with a permanent magnet moving system. The core of the device, where the fluorescence-based bioassay took place, was a novel plastic chip equipped with ten separate microfluidic channels which allowed the simultaneous measurement of different immunosuppressants. Each microfluidic channel was integrated with an illumination system via an optical fibre and then coupled with a photodiode through an optical filter. Finally, the bottom of the chip was connected with an array of ten permanent magnets (Fig. 1). Details on the design, optimisation and final integration of the device are given in the ‘Results and discussion’ section.

### Materials and protocols

#### Fluorescent magnetic particles

Commercial magnetic 300 nm diameter particles (Estapor®, Merck Millipore) with a polystyrene core were used. The polystyrene particles were decorated with ferrite nanograins (ca. 8–10 nm) that conferred superparamagnetic properties. They were also functionalised with carboxylic groups on the surface, which were needed for further bioconjugation. These non-fluorescent particles were doped with a hydrophobic boron-dipyrromethane BODIPY 641 dye by a swelling procedure [31], yielding brilliant fluorescent beads (λ_ex_^max^ = 640 nm; λ_em_^max^ = 680 nm) despite the dense ferrite shell on the surface.

#### Immunosuppressants and their derivatives

Mycophenolic acid (MPA), cyclosporine A (CsA) and tacrolimus powders used as standards were purchased from Abcam. The cyclosporine A carboxylic acid derivative (CsA-O-CO_2_H) was prepared as described in [25]. The following antibodies were used: polyclonal sheep anti-mycophenolic acid IgG antibody (Abcam) and monoclonal mouse anti-cyclosporine A IgG1 antibody (clone CSZ2.22) (Abcam).

#### Other reagents and solutions

1-Ethyl-(*N,N*-dimethylamino)propylcarbodiimide hydrochloride (EDC) and *N*-hydroxysuccinimide (NHS), Tween 20, reagents for buffer preparation and sodium dodecyl sulphate (SDS) solution were all from Sigma and Lipofundin MCT/LCT 20% from B. Braun Melsungen.

#### Immobilisation of antibodies onto the magnetic fluorescent particles

For the immobilisation of antibodies onto the fluorescent magnetic particles (FMPs), the carboxylic groups on the FMPs were activated by means of EDC (40 mg/mL in MES buffer) for 10 min. After separation with an external permanent magnet the activated FMPs were incubated with the immunosuppressant-specific antibody (10 μg/mL) for 2 h. After separation, the FMPs were washed 3 times with phosphate-buffered saline (PBS), blocked with Pierce protein-free blocking buffer (ThermoFisher Scientific, Monza, Italy) and washed again with PBS. After washing, the coated FMPs were stored at 4°C.

#### Immobilisation of immunosuppressants onto the microfluidic channels of the chip

The channels of the chip were modified with MPA or the CsA-O-CO_2_H derivative via carbodiimide crosslinking chemistry [25]. The carboxylic groups of the antigen were firstly activated by a freshly prepared aqueous solution containing 200 mM EDC and 50 mM NHS and then flowed through the channels for 1 h using a peristaltic pump (Gilson, Milan, Italy). After immobilisation, the channels were washed with PBS and blocked with undiluted protein-free blocking buffer to prevent any potential non-specific adsorption.

#### Assay general protocol

**Fig. 2:**
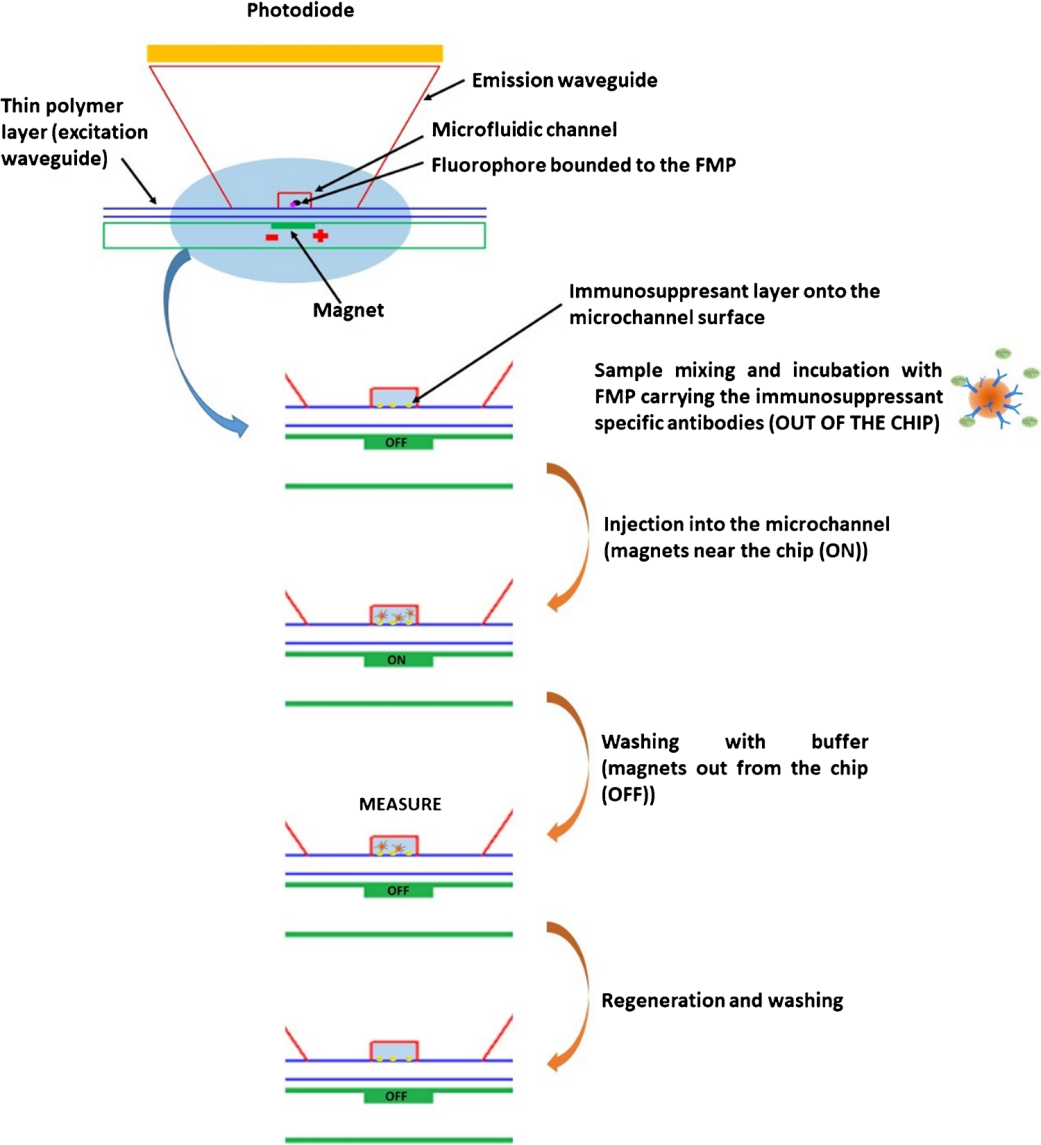
Scheme of the different steps of the on-chip immunoassay

The general protocol of the assay for the determination of the immunosuppressants inside the integrated optical device is detailed in Fig. 2. The perfusate/microdialysate sample containing the immunosuppressants was mixed and incubated with the FMPs carrying the immunosuppressant-specific antibodies outside the measuring chip, with the use of a proper mixing chip; the mixed sample/FMPs was then pumped sequentially into the different microfluidic channels of the chip. During this step, the magnets at the bottom of the channels were moved close to the chip and the antibodies onto the particles that were not bound to the immunosuppressant, interacted with the immunosuppressant immobilised onto the surface of the microchannel. The magnets were then moved away from the chip and washing with buffer was conducted in each microfluidic channel. During this step, the antibodies, and consequently the FMPs, which did not interact with the analyte onto the surface of the channels were washed away. Finally, the fluorescence measurement was performed, simultaneously onto all the microfluidic channels.

After the measurement, regeneration of the channels surface was carried out, followed by washing with buffer and pumping of air inside all the fluidics in order to assure the same starting conditions for each measurement (each sample).

Details on the fluidic procedures used for the final assays conducted inside the integrated optical device are described in the following section.

#### Spiked samples and real microdialysate samples

CsA and MPA were spiked into 20% Lipofundin MCT/LCT (20%) in isotonic saline solution at different concentrations (range: 0–30 ng/mL for MPA and 0–10 ng/mL for CsA). Real microdialysate samples were collected from ex vivo microdialysis of whole blood samples of immunosuppressed patients by using a MicroEye® catheter (9,000 Da cut-off; Probe Scientific, Bedford, UK) combined with a Technic I syringe pump (AMV technics, Předklášteří, Czech Republic) set to a flow rate of 1.6 μL/min (see [25] for more details) and using 20% Lipofundin MCT/LCT (20%) in isotonic saline solution as perfusate.

#### Fluidic protocol for the sample analysis in the integrated device

The microfluidic chip, functionalised with NH_2_ groups, was coated with MPA in four channels and with CsA-O-CO_2_H in other four by using a peristaltic pump before its loading into the instrument. The CsA- and MPA-coated chip was then loaded into the optical device for its connection to the fluidic system. The modified surface of the channels was subsequently exposed to solutions containing the anti-CsA and anti-MPA antibody-coated FMPs mixed and incubated with Lipofundin (20% in PBS), with the real samples (final dilution 1:1) or with different concentrations of CsA and MPA diluted in 20% Lipofundin.

The following fluidic procedure was used for the binding phase of the assay:

1. Filling of the fluidic line from the solution vial to the fluidic switch used for the channel selection (60 s);
2. Sequential filling of the channels (10 s per channel);
3. Re-filling of the channels (5 s per channel, repeated 3 times).

After the first filling of the ten microfluidic channels and between each re-filling step, the array of permanent magnets was actuated for 30 s. By following this fluidic/magnetic procedure, the sample was incubated in each channel for a total time of 5 min. At the end of the assay procedure, the channels were washed with a procedure consisting of 15 s of buffer flowing per channel repeated 5 times. The total assay time comprising binding and washing was 16 min.

For the regeneration step, after filling the fluidic line from the solution vial to the switch with the regeneration solution (SDS 0.25%, pH 2.5), a volume of 10 μL of this solution was sequentially injected in each microfluidic channel with a sequence of short pulses assuring that its permanence in each channel was 60 s. The washing step after the regeneration was conducted as reported before for the washing after the binding phase.

The fluorescence emitted by the sensing layer immobilised within the microfluidic channels was recorded every 2 s as the average of 8 acquired values, using a photodiode integration time of 250 ms.

## Results and discussion

### Design and implementation of the optical device

As illustrated in ‘The optical device’, the core of the optical device was the microfluidic chip with the optical components designed and optimised to achieve the necessary performances for its use in the analysis of clinical samples. The different components of the device together with their design and implementation are illustrated in the following sections.

#### The microfluidic optical chip

**Fig. 3:**
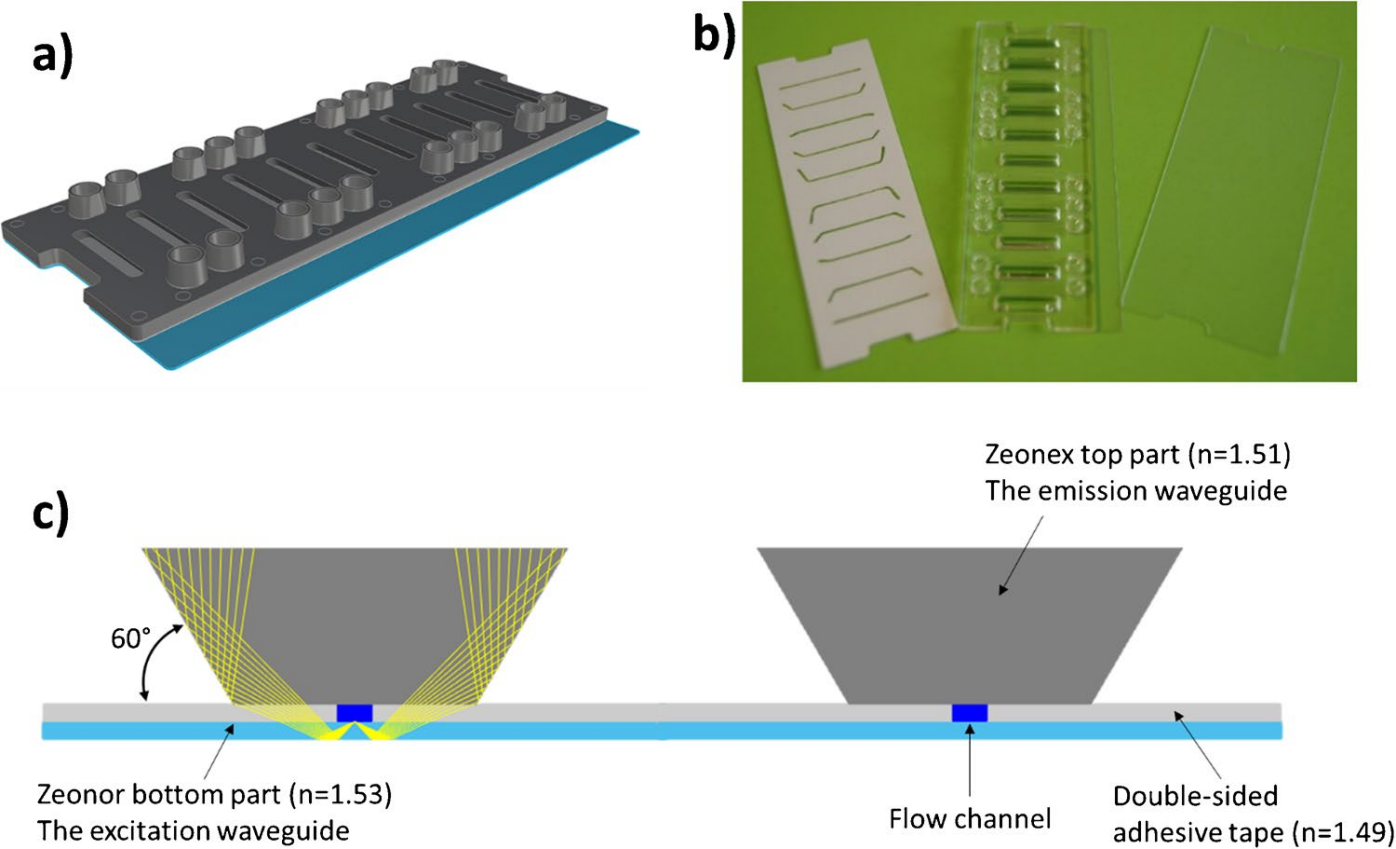
**a** 3D scheme of the microfluidic chip. **b** The three components of the optical chip. From left to right: double-sided adhesive tape with laser-cut microchannels, molded Zeonex^®^ top part with integrated fluidic ports and Zeonor^®^ bottom foil. **c** Magnified view of the cross section of the microfluidic chip depicting two of the channels (the yellow lines represent the optical path of the fluorescence rays)

The 3D scheme of the microfluidic optical chip is shown in Fig. 3a. It comprised three parts (Fig. 3b): the Zeonor^®^ bottom foil (dimensions 75.3 mm × 30.4 mm × 0.19 mm, refractive index *n*=1.53), the injection-molded Zeonex^®^ top part (75.3 mm × 25.4 mm × 1.5 mm, *n*=1.51) and the double-sided adhesive tape (75.3 mm × 25.4 mm × 0.14 mm, n=1.49), where the microchannels were manufactured by laser cutting. The flow channel depth, fixed by the adhesive tape thickness, is 140 μm, the width is 500 μm and the length of the straight part is 14 mm.

The upper part of the Zeonor^®^ foil was functionalised with amino groups to allow the immobilisation of the MPA and CsA-O-CO_2_H derivatives (see ‘Materials and protocols’) so that, concerning the flow channel, the bioactive surface of the channel was only the bottom one. In addition, compared to the Zeonex^®^ top part and the adhesive tape, the Zeonor^®^ material of the bottom part of the chip had a higher refractive index.

By this means, the Zeonor^®^ foil could be used as an optical waveguide element due to its higher refractive index: the excitation light, coming from an external source (see next subsection), could propagate inside the foil by total internal reflection (TIR) and could excite, by its evanescent field, the fluorescent sensing layer immobilised on the bottom of the microfluidic channels. A scheme of the cross section of the chip is depicted in Fig. 3c, where the optical path of the fluorescence rays is sketched in yellow. The Zeonex^®^ part topping each microfluidic channel was shaped so that its side walls form an angle of 60° with the horizontal line. As the fluorescence was not isotropic, due to the closeness of the fluorophore to the interface [32], the emission signal coming from the FMPs bound to the biosensing layer was mainly coupled inside the denser plastic foil with an angle close to the critical angle at the interface Zeonor^®^-buffer solution (~ 61°). Thus, after a reflection at the Zeonor^®^-air interface, the fluorescence signal mainly hit the top part of the chip in normal direction, thanks to the believed lateral sides. In this way, the fluorescence collection by the photodetectors located on top of the chip (see next subsection) was optimised.

#### The optoelectronic excitation and detection module

The excitation light is generated by a 30 mW TEM00 CW diode-pumped solid-state laser emitting at 589 nm (CNI MGL-III-589, Changchun New Industries Optoelectronics Technology) coupled to a bundle of eleven fibres (Molex Polymicro FVP150165195, high-OH silica/silica core diameter 150 μm, cladding diameter 165 μm, polyimide buffer 195 μm, NA 0.22). The laser side of the bundle is terminated in circular shape and mounted on a micrometric alignment holder (Thorlabs K5X1 5-Axis Locking Kinematic Mount) facing the laser source. On the other end, ten fibres are directly faced to the chip by butt coupling them to the lateral side of the Zeonor^®^ foil and aligned with the direction of the microfluidic channel (Fig. S1b and c). The eleventh fibre is interfaced to an external photodiode (Thorlabs SM05PD1B large area mounted silicon photodiode) for compensating possible laser power fluctuations. The rendering of the excitation module of the POCT device together with details of the fibre coupling and of the microfluidic chip is given in Fig. S1 of the supplementary information.

The detection module, which allowed to completely segregate the fluorescence generated in each of the ten microchannels, was formed by an array of ten optical filters (Schott RG645, coloured glass long pass filters) placed within a black anodised aluminium filter holder for straylight blocking and elimination of the cross-talk between adjacent filters, ten photodetectors (one for each microchannel) and the printed circuit board (PCB) equipped with spring-loaded connectors for the electrical connections with the photodetectors and with SMA RF connectors for the electronic boards. An exploded view of the detection module is provided in the supplementary information (Fig. S2a). The photodetectors were thin film nip-diodes made of hydrogenated amorphous silicon (a-Si:H) [33]. The main advantages of these a-Si:H-based thin film photodetectors were their low dark current and variable spectral response, the possibility to deposit detector elements at low temperature directly onto different superstrates (e.g. glass) or substrates (e.g. metal foil and silicon wafer), in particular with respect to microfluidic components, and their low mass production cost, which made them a good solution for the detection of very small radiation levels originating from low concentration fluorophore emission [34]. One of the ten photodiode series used in this system is depicted in Fig. S2b. The active area (36.3 mm^2^) and the two pads used for the electrical connections with the spring-loaded pins placed on the PCB board are visible in the pictured a-Si:H photodiode.

#### Permanent magnet moving system

A module with a permanent magnet array equipped with vertical motion was specifically developed for the device (Fig. S3a). The module was designed to be compatible with the integrated system and consists of a holder with 10 small neodymium-iron-boron permanent magnets (PM) (Supermagnete, Gottmadingen, Germany) with dimensions 10 mm × 3 mm × 2 mm and a micro servo motor (5087MH HV, Hitec, USA), controlled by PC that allowed the vertical motion of the magnets. Here, the PM array was properly designed to have each magnet in correspondence with each microfluidic channel of the optical chip (Fig. S3b) and the typical actuation time of the motor was of the order of 0.1–0.5 s.

#### The fluidic system

Commercial pumps, valves and flow sensors (Fluigent^®^) were used for the fabrication of the fluidics module. The technology provided by Fluigent^®^ was based on a pressure-driven system that enabled the pulse-less flow actuation of fluids by pressure regulation.

**Fig. 4:**
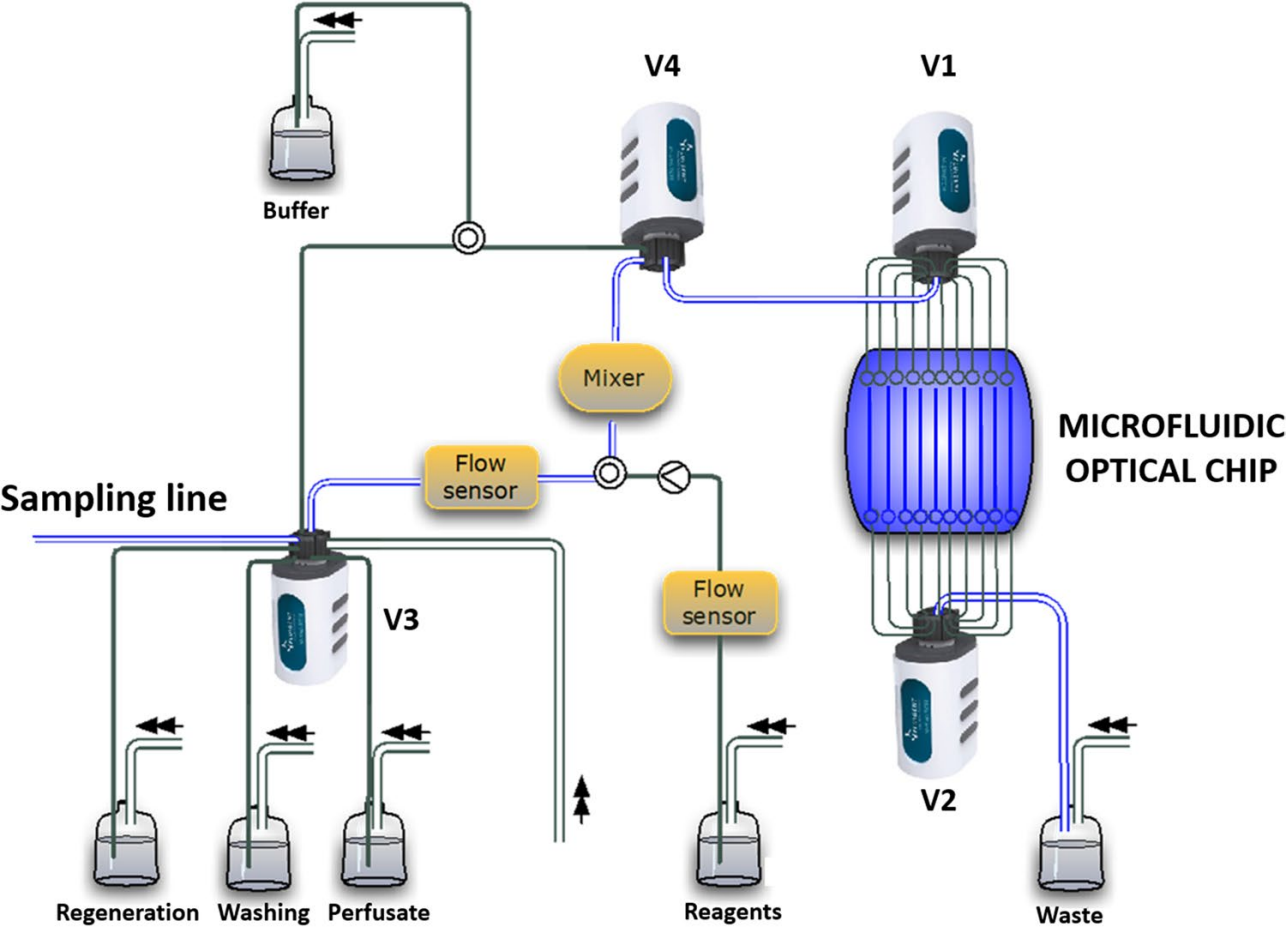
Schematic view of the fluidic system based on Fluigent® pressure-driven microfluidics pumps

The scheme of the fluidic system is depicted in the diagram of Fig. 4. The selection of each channel and the sequential filling of the ten channels of the microfluidic chip was controlled by two bidirectional 11-port/10-way valves (Fluigent^®^ M-Switch™), indicated with V1 and V2 in Fig. 4. Two additional valves were used for the selection of the sample from the patient line or of the reagent/washing/perfusate lines (V3) and the selection of the sample/chemicals line or of the buffer line (V4). Two flow sensors (Fluigent^®^ Flow Unit, 0–80 μL/min flow rate range), located in upstream of the mixer, accurately checked the flow rate. Additionally, the pressurisation of the waste vial could allow the forward and reverse flow inside the chip microchannels and inside the mixer. A suitable mixer, for mixing and incubating the sample with the antibody-coated FMPs outside the measuring chip, was specifically developed by microfluidic ChipShop and fabricated by injection molding in transparent thermoplastic polymer (Topas®). Its scheme with inlet and outlet channels on both sides, two equal 40 μL reaction chambers and a herringbone structure to facilitate mixing is provided in the supplementary information (Fig. S4).

#### The software

A commercial tablet PC (Motion^®^ C5te, Motion Computing Inc) specifically designed for use in hospital environment was chosen as user interface. Communication with the POCT device was possible through a physical connection via either USB or ethernet ports. A 32-bit ARM^®^ Cortex^®^-M0 PSoC^®^ 4 microcontroller was used for the electronic main boards while Python^®^ was adopted for the firmware development along with the libraries Thinker, Serial and Matplotlib. For the control of fluidics components, the firmware was based on the Fluigent^*®*^ MFCS™ Series SDK, which enabled access to low-level control of the components.

#### The chip loading-engaging module

The connection of the microfluidic optical chip with the microfluidic module was performed by means of a microfluidic manifold (dimensions: 83 mm in length, 36 mm in width and 13 mm in height) that engaged the 20 mini-Luer connections of the chip by exerting a suitable pressure (Fig. S5a). The manifold contained the optical filters and photodiodes and it was moved by a stepper motor linear actuator (L5918S2008-T10X2, Nanotec) that provided the necessary torque for the chip-manifold engaging (Fig. S5b). The chip loading was performed manually thanks to a sliding loading tray that allowed the firm positioning of the chip and the accurate alignment of the chip with the fluidic manifold and consequently with the photodiodes. The manifold also ensured the automatic alignment of the chip with the excitation fibres.

### Component optimisation and measurements with the integrated device

The correct working of the FMPs regarding their interaction with the sensing layer and their capability to accelerate the assay was investigated by coupling the optical chip with the permanent magnet moving system shown in the supplementary information (Fig. S3a) and then evaluating the fluorescence associated with the microfluidic channels by acquiring an image of the complete channel with an inverted fluorescence microscope Zeiss AxioObserver.Z1 (5× objective, λ_ex_ 625 nm, integration time 3 s).

A suspension of anti-MPA antibody-coated FMPs was pumped into two microfluidic channels coated with tacrolimus or MPA, respectively, using a peristaltic pump at a flow rate of 4 μL/min according to the following protocol:

- Filling the channels with the FMPs suspension;
- Stopping the flow for 30 seconds;
- Raising the permanent magnet array until contacting the chip;
- Allowing interaction with the microchannel surface for an established time;
- Lowering of the magnet array and flowing off the FMPs for 30 s at 4 μL/min.

These steps were repeated three times and then the channels were washed with PBST (PBS containing 0.05% of Tween 20) for 4 min at 200 μL/min.

The images were analysed by using the microscope software and evaluating the densitometric value (average grey level of the image pixels) over the selected area corresponding to the whole channel. The average densitometric values were then evaluated by subtracting the background value corresponding to a blank and not-used channel.

Two different interaction times, 30 min and 5 min, were used to verify the capability to perform the assay in shorter times and thus increase the frequency of measurements, which is an essential aspect in TDM. As shown in Table 1, in the absence of a magnetic trap the fluorescence intensity decreased with decreasing the interaction time, but the specific/non-specific ratio did not change. Thanks to the magnetic trapping achieved with the 10-magnet array, the fluorescence intensity from the channel increased two-fold for the same interaction time (Table 1) and, more interestingly, a higher specific/non-specific ratio was obtained, demonstrating the benefits derived from the use of the magnet array. The increased specific/non-specific ratio could be due to a combination of factors, including the decreased interaction time which fosters the specific binding with respect to the non-specific interaction, the different diffusion rates, and the affinity balance between the specific elements of the immunoassay.

**Table 1:** Fluorescence intensity in arbitrary units, given as densiometric value, on two different microfluidic channels coated with tacrolimus and MPA, respectively, measured after the injection of anti-MPA antibody-coated magnetic particles for two different interaction times, with and without using the 10-magnet array

Immunosuppressant immobilised on the channel surface	Interaction time 30 min/No magnet	Interaction time 5 min/No magnet	Interaction time 5 min/10-magnet array
Tacrolimus (non-specific)	19.3	5.4	11.7
MPA (specific)	39.1	10.7	68.2
Specific/non- specific ratio	2.0	2.0	5.8

**Fig. 5:**
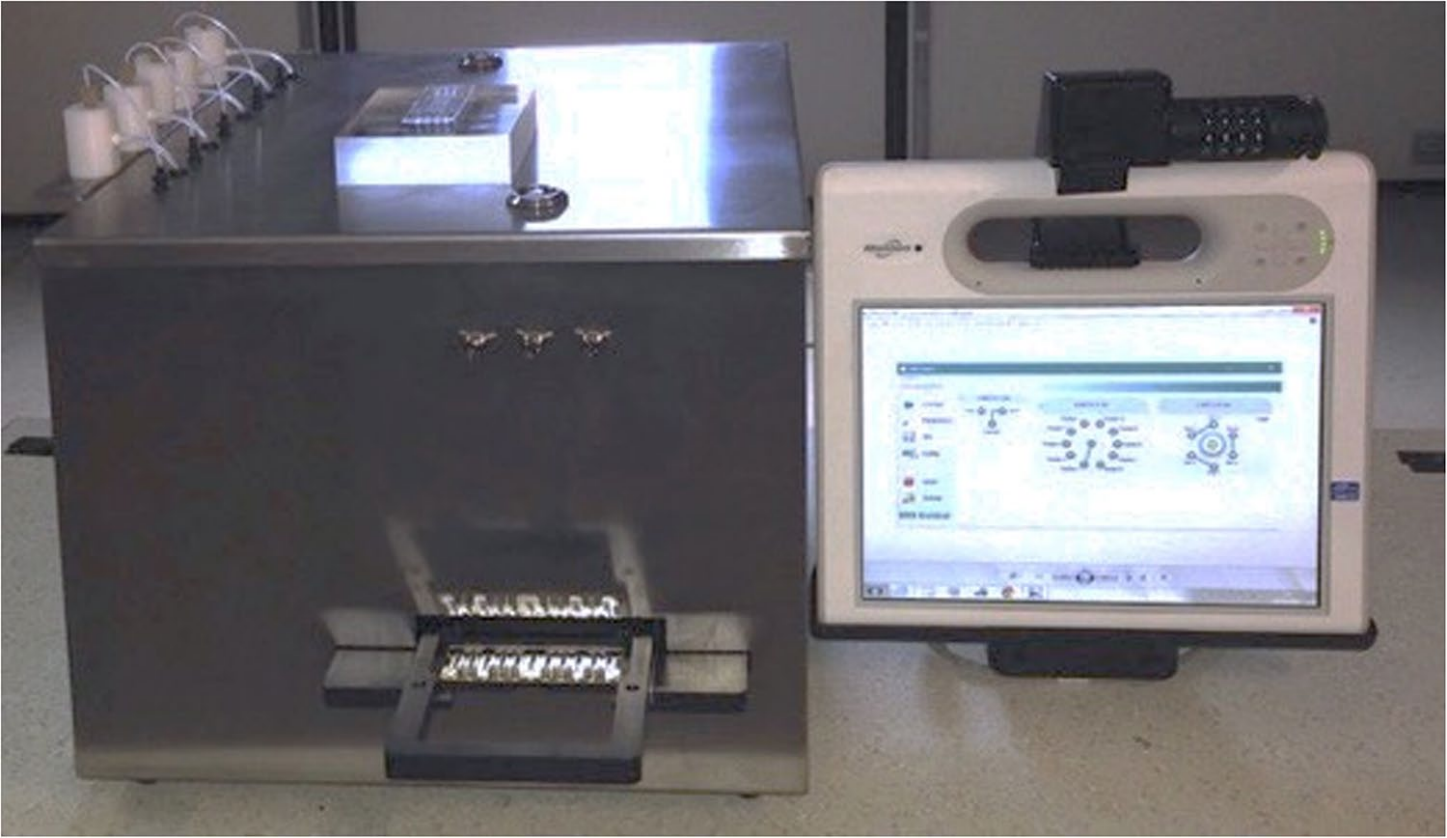
Actual optical integrated device interfaced to the tablet PC

Figure 5 shows the realised integrated optical device (dimensions: 28 cm in height, 36 cm in length and 45 cm in width 45 cm; weight: 20 kg). A thorough characterisation of its performance was carried out to ensure the reliability of the device before performing any assay.

The optical chip designed with the excitation provided by the evanescent wave propagating along the Zeonor foil allowed the geometric separation of the excitation and emission light paths, but any influence coming from the laser-scattered excitation light had to be avoided in order to allow highly sensitive fluorescence measurements. Because of the small distance of the photodetectors from the chip and, therefore, from any potential scattering sources arriving from different directions, the scattered excitation stray light could not be filtered using interference filters exhibiting a strong angular dependency and thus long-pass absorption glass filters were preferable [35]. These filters were inexpensive and exhibited angle-independent spectral filtering properties. As shown in Fig. S2a in the supplementary information, a black filter holder was used to further shield the photodiodes from any other additional unwanted scattered light, and to avoid that the fluorescence generated in one microfluidic channel could reach the adjacent photodetectors, each of them dedicated to the detection of the fluorescence coming from the adjacent channels.

**Fig. 6:**
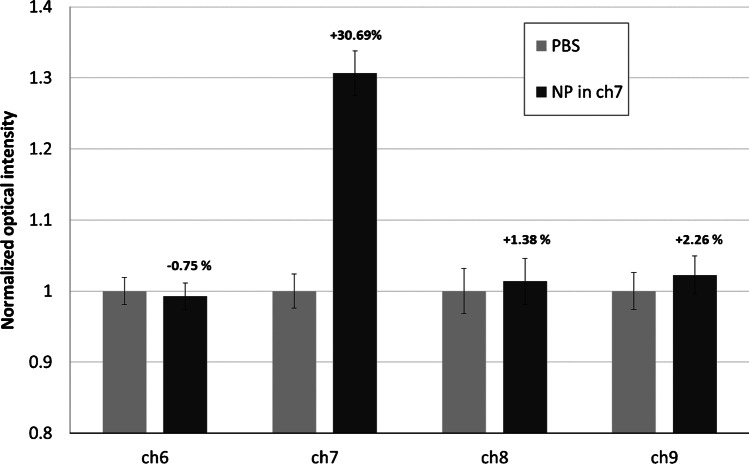
Evaluation of the crosstalk between the microfluidic/optical detection channels (ch). Light gray bars: normalized fluorescence from all the channels filled with PBS; dark gray bars: fluorescence from the FMP attached to the bottom surface (Zeonor^®^) of ch7 microfluidic channel

The absence of any crosstalk between adjacent microfluidic channels was carefully verified. For this purpose, one channel (channel 7) of the chip was modified with MPA via the carbodiimide coupling method (‘Materials and protocols’) and the signals coming from channel 7 and from the adjacent channels (channels 6, 8 and 9) were acquired after their filling with PBS (light grey bars in Fig. 6) and set to 1. Channel 7 was then filled with the FMPs modified with anti-MPA antibody for 30 min. The signals acquired after the subsequent channel washing were normalized to the original signals when all channels were filled with only PBS (no fluorescence) (dark grey bars in Fig. 6). The relative variation of the signals in the four channels is shown in Fig. 6: the observed variations within the error bars in channels 6, 8 and 9 and the strongly increased fluorescence signal of FMPs-loaded channel 7 excluded any fluorescence crosstalk between adjacent channels of the chip.

The optoelectronic and mechanical stability of the device was verified by measuring the signal coming from a chip with eight channels filled with buffer for 2 h, without performing any fluidic procedure. In this way, the only measured contribution arised from the scattered excitation light, and any fluctuations in the detected signals had to be ascribed to fluctuations of the optoelectronics (source and/or detector fluctuations) and/or to micrometre movements/misalignments of the mechanical parts. As shown in Table 2, a standard deviation less than 2.5% was observed for all the microchannels except for channel 6 (ch6), where the standard deviation was 5.2%, confirming the good stability of the device.

**Table 2:** Statistical analysis of the signals acquired in stable fluidic conditions on eight channels of the chip for 2 h

	ch2	ch3	ch4	ch5	ch6	ch7	ch8	ch9
Mean value of the signal	290350	345750	296940	185060	154340	150050	127990	152950
Standard deviation	6540	7040	6680	1970	7950	865	420	1850
Relative error	2.3%	2.0%	2.2%	1.1%	5.2%	0.6%	0.3%	1.2%

**Fig. 7:**
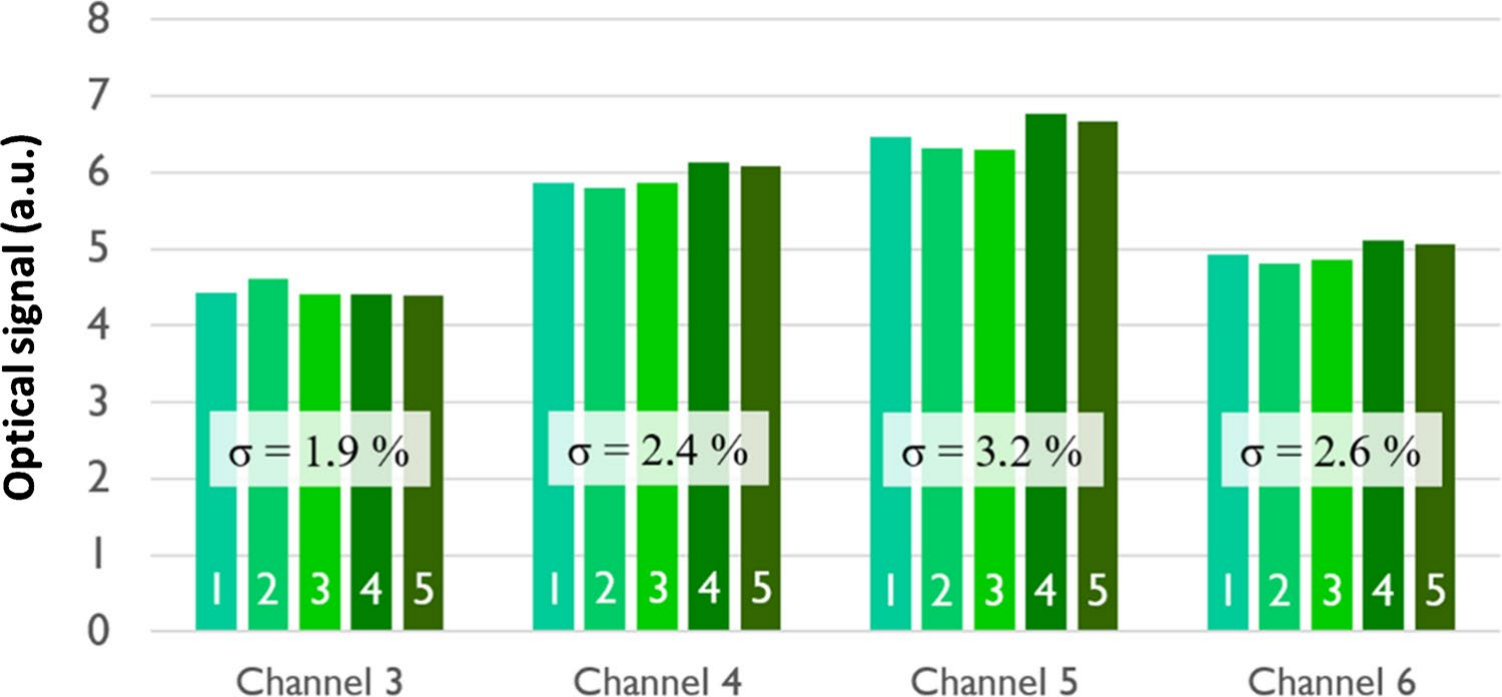
Detected optical signals from four different channels filled with PBS buffer after successively loading the chip inside the optical device five times. The standard deviations (σ) of the five different acquired signals are indicated

The reliability of the chip loading-engaging procedure described above was verified by loading and removing the same chip five times and comparing the acquired signal coming from four microfluidic channels (channels 3–6) filled with PBS buffer. The measured signals summarised in Fig. 7, with their average and the standard deviation values, were characterised by very low fluctuations demonstrating the good repeatability of the adopted procedure. The absolute difference in optical signals between the different channels is probably due to a non-homogenous alignment of the eleven fibres bundle and the laser excitation beam and to possible different losses suffered by different fibres. The consequence is that the fibres do not carry exactly the same optical power, but the illumination of each channel is sufficiently stable in time, so that the signals of the ten channels can be normalized after a calibration.

Lipofundin samples spiked with CsA and MPA and real microdialysate samples, resulting from ex vivo microdialysis of whole blood samples from immunosuppressed patients, were tested with the developed optical device.

A calibration curve for CsA was obtained by using 20% Lipofundin spiked with different concentrations of CsA. The different solutions were tested sequentially with a regeneration step between each assay. Three assay procedures were conducted for each concentration; the blank sample contained only 20% Lipofundin and corresponded to the maximum signal with no binding inhibition, as tested at the beginning of the series and every 5 measurements with other concentrations. The different concentrations were tested with a random distribution in the series including the measurements of the real microdialysate samples.

A total of 21 regeneration cycles was conducted with a recovery signal ranging from 75 to 90% with respect to the previous background point. This recovery variation did not affect the reproducibility of the signal shift for each concentration and consequently the B_x_/B_0_ ratio indicating the binding inhibition (ratio among the signal obtained with a particular concentration of the antigen and that without the antigen).

**Fig. 8:**
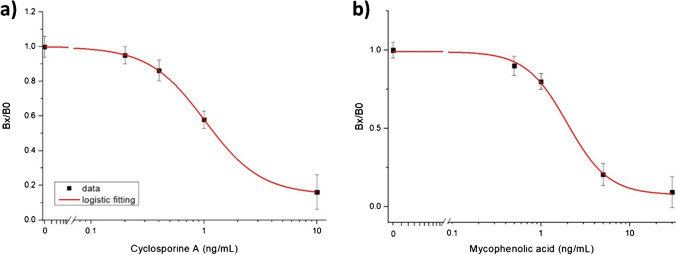
**a** Calibration curve for CsA obtained with the optical device using FMPs coated with anti-CsA antibody and CsA diluted in 20% Lipofundin. **b** Calibration curve for MPA obtained with the optical device by using FMPs coated with anti-MPA antibody and MPA diluted in 20% Lipofundin

The calibration curve for CsA, obtained with this series of measurements, is shown in Fig. 8a. With the parameters of the best logistic fitting curve (*A*_1_=0.999, *A*_2_=0.146, *x*_0_=1.02, *p*=1.75, where *A*_1_ and *A*_2_ are the asymptotes of the fit, *x*_0_ is the value of the CsA concentration for which the fluorescence signal is equal to the 50% of the dynamic range and *p* gives the slope of the curve for *x*=*x*_0_; adjusted *R*-squared = 0.9998) and considering the error on the zero concentration a limit of detection (LOD; calculated from the logistic fitting by using three times the error on the blank) of 0.48 ng/mL was obtained [36]. The working range, evaluated by considering the concentration range between 10 and 90% of the difference between the upper and lower asymptote, the so-called dynamic range [37], resulted in 0.57–10 ng/mL. To evaluate the reproducibility, the coefficient of variation (CV%) of each tested concentration was calculated by considering the standard deviation resulting from the different measurements conducted on each concentration: by averaging the resulting CV%, a value of 7% was obtained which demonstrates a good reproducibility of the assay.

Two real microdialysate samples from patients were also tested and their CsA concentrations were calculated from the calibration fitting curve of Fig. 8a. Concentrations of 0.50 ± 0.03 ng/mL and 0.57 ± 0.04 ng/mL were obtained for the two samples, which were coherent with the expected concentration of free CsA in the microdialysate samples, according to the total CsA-to-CsA in microdialysate ratio reported in ref [25].

The same procedure was adopted to obtain the calibration curve for MPA (Fig. 8b). In this case, the signals from the four MPA-modified channels were considered.

With the parameters of the logistic fitting curve (*A*_1_=0.993, *A*_2_=0.076, *x*_0_=1.96, *p*=1.87; adjusted *R*-squared = 0.9918) and considering the error on the blank, a LOD of 0.79 ng/mL was calculated with an average CV of 7% and a working range of 0.87–17 ng/mL. The concentration of MPA detected in the two real microdialysate samples (3.7 ng/mL and 30 ng/mL, respectively) showed a higher variability than that of CsA. Due to the better dialysability of MPA and the broader concentration range of MPA [25], these significantly higher concentrations were reasonable.

## Conclusions

A fluorescence-based POCT device was developed for the simultaneous automatic measurement of a mixture of immunosuppressants towards its application in TDM. This capability was achieved, thanks to the development of a 10-channel microfluidic chip, designed to optimize the collection of the laser-induced fluorescence from a bioaffinity layer after interacting with the immunosuppressant of interest in the presence of tailored fluoromagnetic particles. The high efficiency of the signal collection allowed to cover the working range of the immunosuppressants requested for clinical applications with promising results for CsA and MPA while the use of FMPs led to a significant reduction of the interaction time of the analyte with the biosensing layer, yielding an overall assay time of just 16 min. There is still some work to be done, as the achieved working ranges do not meet completely the requirements of the clinicians: even if sufficiently sensitive to the low levels of drug concentrations in microdialysates, the ranges have to be extended to higher values. This is particularly true for MPA which is highly dialyzable due to its high polarity and low molecular weight and for which values as high as many tens of ng/mL have been found in kidney transplanted patients [25]. The microfluidic circuit integrated in the device allowed the automatic sample handling, from the mixing with the necessary chemicals to the interaction with the biosensing layer. All these features make the developed unit a potential POCT device for immunosuppressant monitoring transplanted patients and for TDM in general, representing an important step forward in the field since the traditional approach of sample withdrawal followed by an analysis in an external laboratory is unable to meet the required short turn-around-time (TAT). Strategies based on sparse blood sampling within longer time windows have been developed for clinical purposes and have been shown to substantially improve the patient’s outcome. Regular pharmacokinetic monitoring requires repetitive blood sampling and our device can offer an essential support to solve this crucial problem with respect to the determination of the correct dosage of the administered drugs. It should also be pointed out that a connection between the novel device microfluidics with the microfluidic line coming out from an intravascular microdialysis catheter could be readily implemented making the quasi-continuous TDM in transplanted patients more than just a dream.

## Supplementary information

ESM 1 (PDF 251 kb)
