# Supplementary material for: An integrated device for fast and sensitive immunosuppressant detection
Source: Anal Bioanal Chem. 2021 Dec 22;414(10):3243–55. doi: 10.1007/s00216-021-03847-x (PMC8956524; doi:10.1007/s00216-021-03847-x)
Supplement: Supplementary file 1 — (PDF 251 kb) [file 216_2021_3847_MOESM1_ESM.pdf]

Supplementary Information

An integrated device for fast and sensitive immunosuppressant detection

Sara Tombelli[1], Cosimo Trono[1]*, Simone Berneschi[1], Chiara Berrettoni[1], Ambra Giannetti[1], Romeo Bernini[2], Gianluca Persichetti[2], Genni Testa[2], Guillermo Orellana[3], Francesca Salis[3], Susanne Weber[4], Peter B. Luppa[4], Giampiero Porro[5], Giovanna Quarto[5], Markus Schubert[6], Marcel Berner[7], Paulo P.Freitas[8], Susana Cardoso[8], Fernando Franco[8], Vânia Silverio[8], Maria Lopez-Martinez[8], Urs Hilbig[9], Kathrin Freudenberger[9], Günter Gauglitz[9], Holger Becker[10], Claudia Gärtner[10], Mark T. O'Connell[11], Francesco Baldini[1]

1) Institute of Applied Physics "Nello Carrara", CNR-IFAC, Via Madonna del Piano 10, 50019 Sesto Fiorentino, Italy
2) Institute for Electromagnetic Sensing of the Environment, CNR-IREA, Via Diocleziano 328, 80124 Napoli, Italy
3) Department of Organic Chemistry, Faculty of Chemistry, Universidad Complutense de Madrid, 28040 Madrid (Spain)
4) Institute of Clinical Chemistry and Pathobiochemistry, Klinikum rechts der Isar, Technische Universität München, Marchioninistrasse 15, W-8000, Munich, Germany -
5) Datamed Srl, Via Grandi 4/6, 20068 - Peschiera Borromeo (Milan), Italy
6) Institute for Photovoltaics and Research Center SCoPE, University of Stuttgart, 70569 Stuttgart, Germany
7) Innovative Pyrotechnik GmbH, Steinwerkstraße 2, 71139 Ehningen, Germany
8) Instituto de Engenharia de Sistemas e Computadores-Microsistemas e Nanotecnologias, R.Alves Redol 9, 1000-027, Lisbon, Portugal
9) Institute for Physical and Theoretical Chemistry, Eberhard Karls University, Auf der Morgenstelle 18, 72076 Tübingen, Germany
10) microfluidic ChipShop GmbH, Stockholmer Str. 20, 07747 Jena, Germany
11) Cornel Medical Limited, 17 Church Walk, St Neots, Cambridgeshire, PE19 1JH, UK

Keywords: immunosuppressant, POCT, fluorescence, cyclosporine A, mycophenolic acid

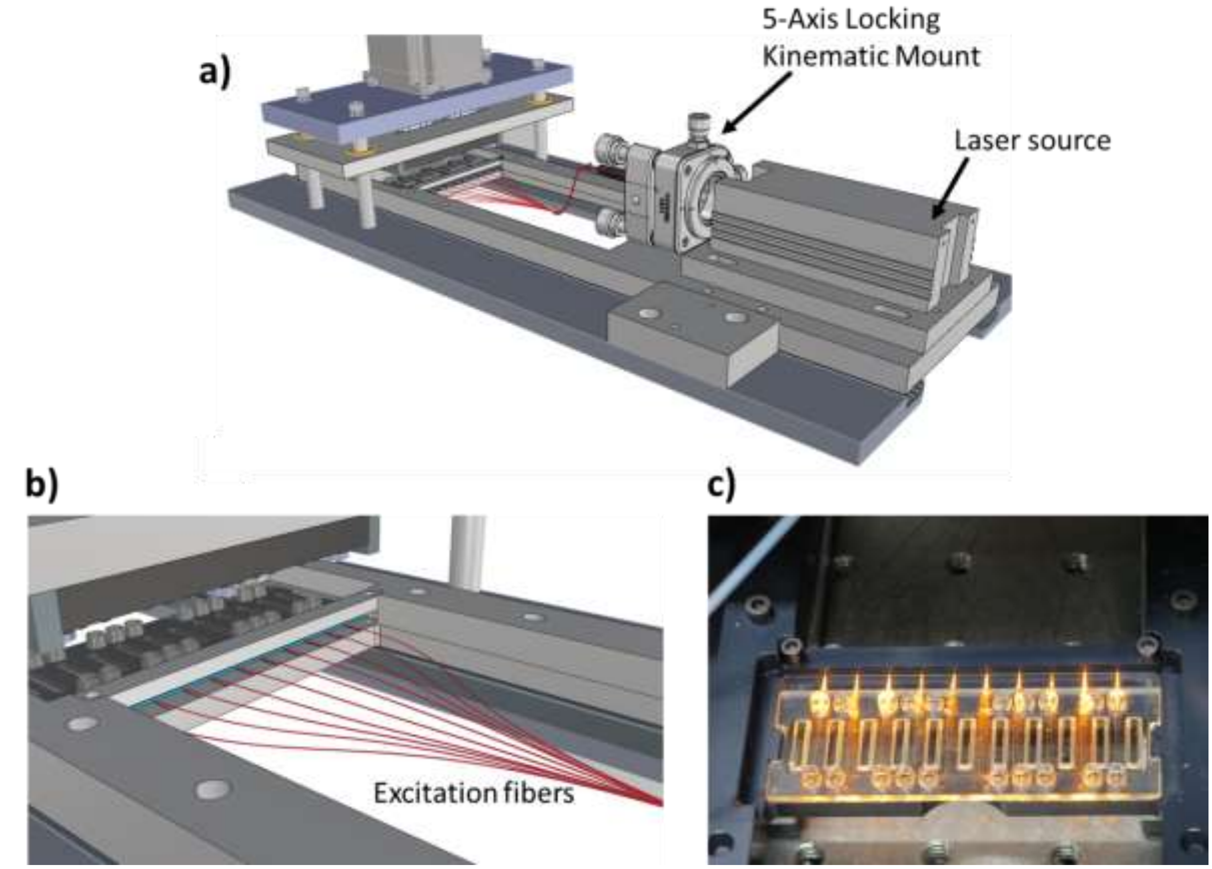

Figure S1. a) Rendering of the device excitation module constituted by the laser source and the kinematic mount for the alignment of the laser beam with the fibre bundle that illuminates the chip channels. b) Rendering of the excitation fibres aligned with the bottom part of the chip made of the Zeonor foil. c) View of the microfluidic chip illuminated by the yellow laser excitation light.

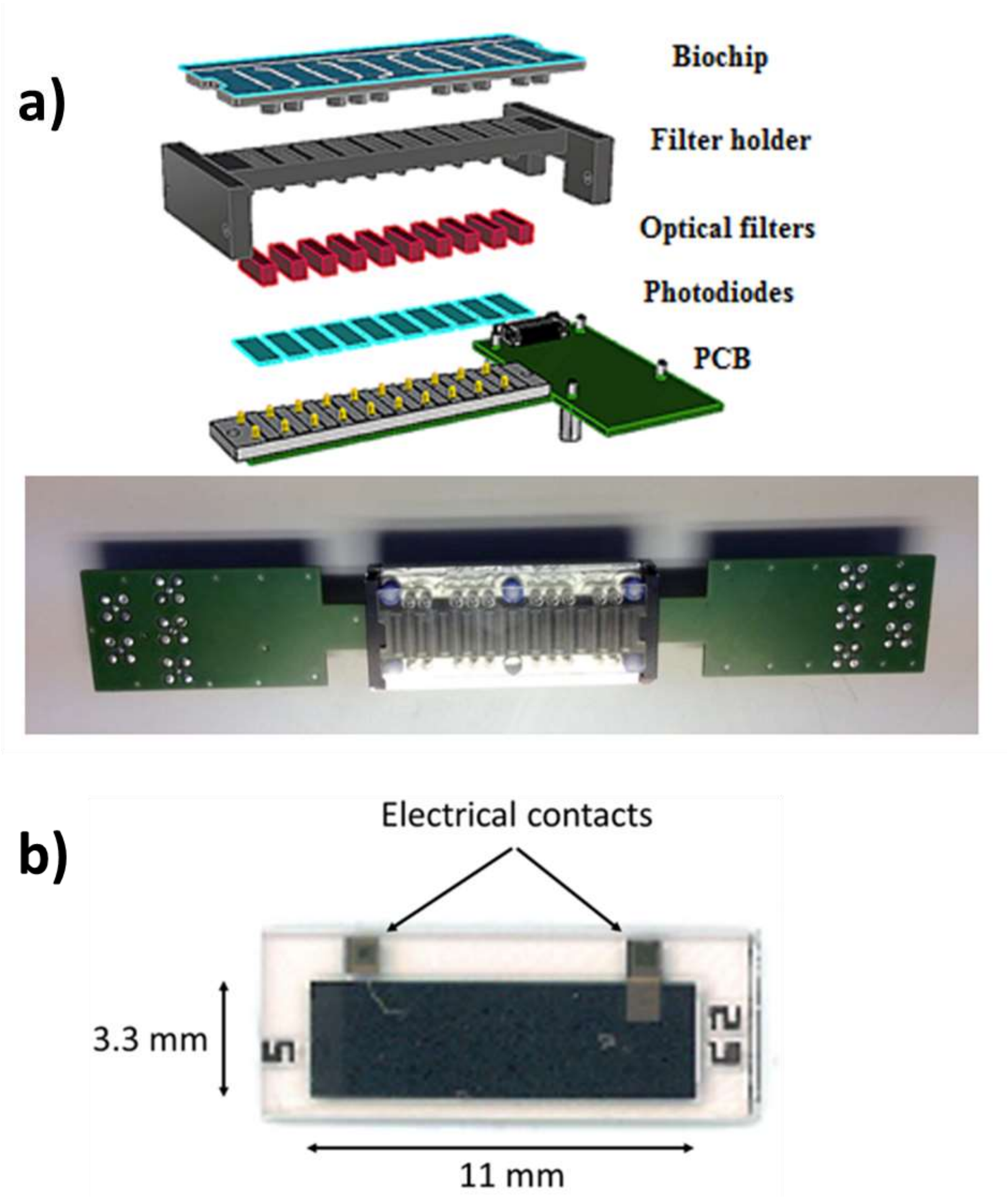

Figure S2. a) Exploded view and picture of the fluorescence detection module assembly. b) The large area amorphous silicon (a-Si:H) photodiode used for the detection of fluorescence coming from each microchannel.

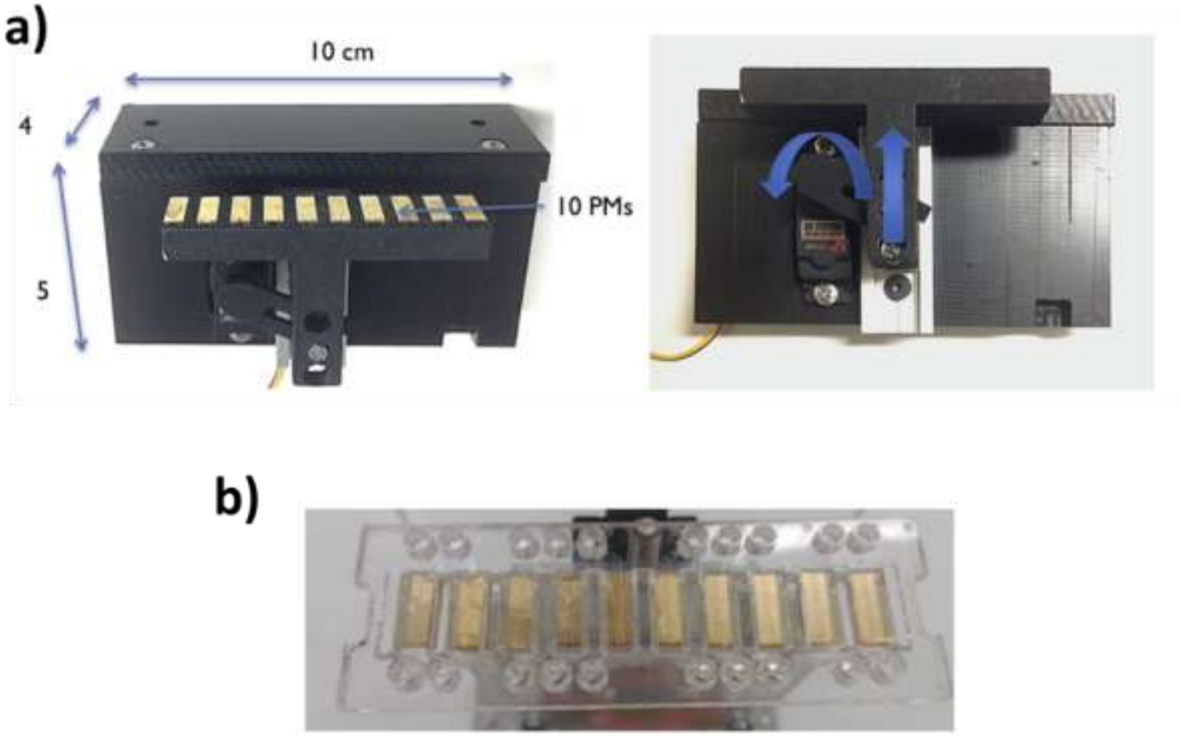

Figure S3. a) The permanent magnet (PM) actuation system. The micro servo motor and the pull up system are visible on the right. b) The ten permanent magnets in contact with the bottom part of the microfluidic chip.

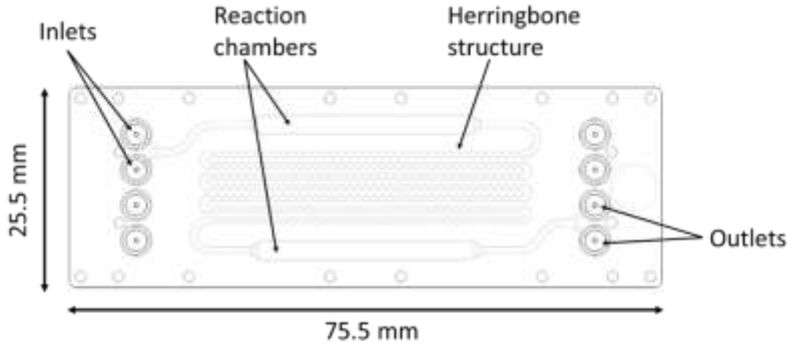

Figure S4. Scheme of the microfluidic ChipShop mixer.

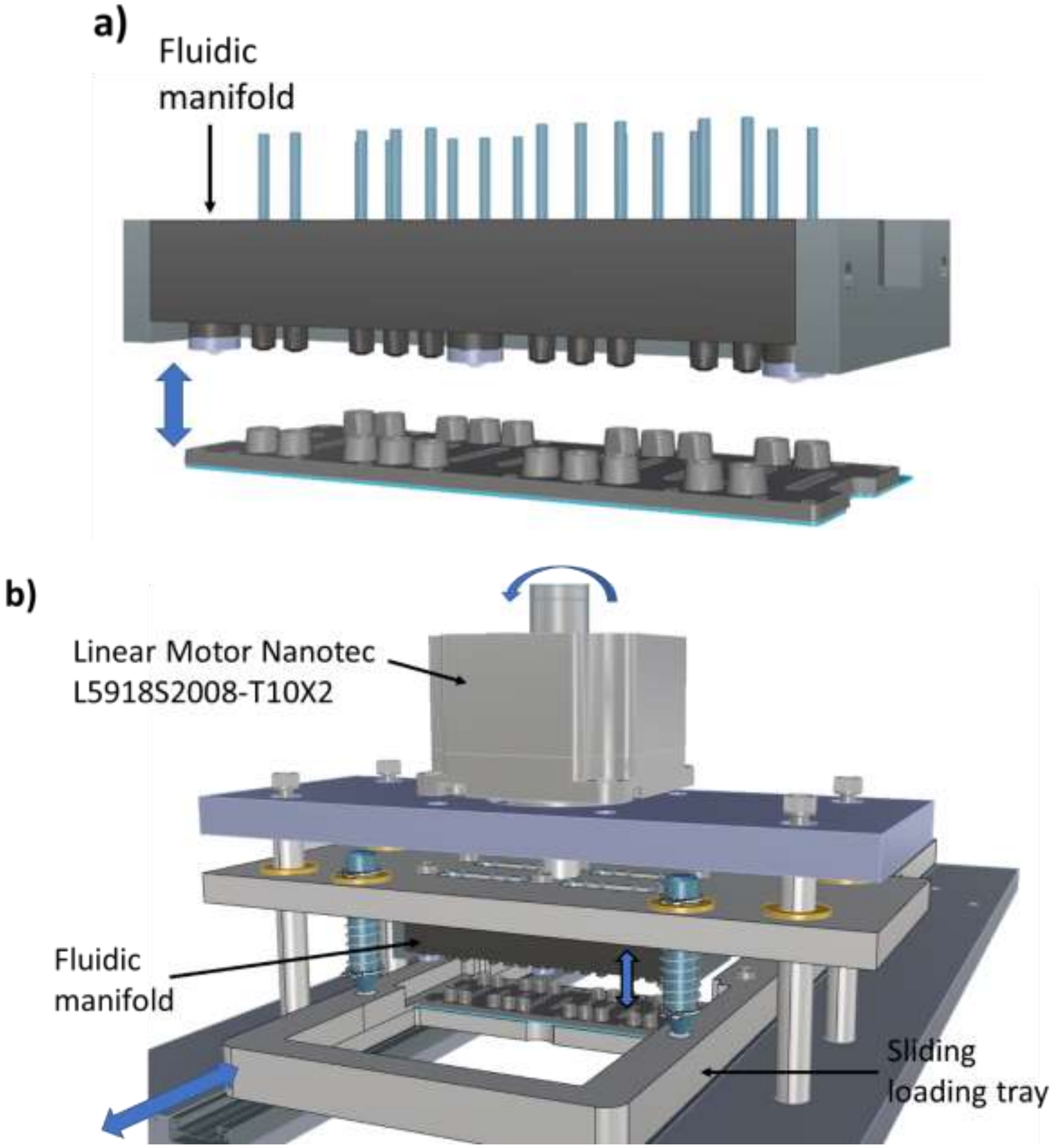

Figure S5. a) Rendering of the fluidic manifold that accomplishes the chip fluidic coupling. b) the motorised actuation system and the chip loading system.
